# Physician-modified fenestration or *in situ* fenestration for preservation of isolated left vertebral artery in thoracic endovascular aortic repair

**DOI:** 10.3389/fcvm.2023.1055549

**Published:** 2023-03-30

**Authors:** Peier Shen, Donglin Li, Ziheng Wu, Yangyan He, Xiaohui Wang, Tao Shang, Qianqian Zhu, Lu Tian, Zhenjiang Li, Hongkun Zhang

**Affiliations:** ^1^Department of Nursing, The First Affiliated Hospital, School of Medicine, Zhejiang University, Hangzhou, China; ^2^Department of Vascular Surgery, The First Affiliated Hospital, School of Medicine, Zhejiang University, Hangzhou, China

**Keywords:** isolated left vertebral artery, physician-modified fenestration, *in situ* fenestration, thoracic endovascular aortic repair, thoracic aortic disease

## Abstract

**Objective:**

To present our experience of preserving the isolated left vertebral artery (ILVA) with physician-modified fenestration (PM-F) or *in situ* fenestration (ISF) during thoracic endovascular aortic repair (TEVAR) for aortic pathologies involving aortic arch.

**Methods:**

This is a single-center, retrospective, observational cohort study. Between June 2016 and December 2021, 9 patients (8 men; median age 60.0 years old) underwent TEVAR with ILVA reconstruction (PM-F, *n* = 6; ISF, *n* = 3) were identified and analyzed.

**Results:**

The technical success rate was 100%. No early (<30 days) death occurred. No aortic rupture, major stroke or spinal cord injury was observed. The median follow up was 38.0 (rang: 1.0–66.0) months. One death occurred at 56 months, while the reason cannot be identified. No aortic rupture, major stroke or spinal cord injury was observed during follow up. No patient required reintervention. Out of the 22 successfully revascularized target vessels, 2 ILVAs were found occluded in 2 patients at 6 months and 7 months, respectively. However, these two patients were asymptomatic.

**Conclusions:**

Our initial experience reveals that PM-F or ISF for ILVA preservation was feasible, safe, and effective during TEVAR for complex thoracic aortic pathologies. However, the patency of preserved ILVA should be improved.

## Introduction

Aortic arch branch variation was common in general population with a proportion approaching 20% (1), while in patients with thoracic aortic disease (TAD), the proportion rises to 33.5%. Isolated left vertebral artery (ILVA) arising directly from the aortic arch was the second most common branch variation with a prevalence of 0.8%–6.6% in TAD (2–5).

It is prevalent that the posterior inferior cerebellar artery was supplied by ILVA (6). Further, in certain aortic arch anomalies, the left common carotid artery (LCCA) does not supply normal blood flow and the ILVA compensates for that (7). Hence, improper management of the ILVA may result in posterior stroke or spinal cord ischemia, especially if the arterial circle of Willis is incomplete. It was reported that a complete circle of Willis was seen in only 27% of Chinese people (8). However, there was no consensus on the indication for preservation of ILVA during TEVAR presently.

The strategies of ILVA reconstruction was still uncertain in current guidelines. ILVA transposition has been used with favorable results (9). Total endovascular reconstruction has the advantages of improved safety and reduced invasiveness. We presented our initial experience and short-term outcomes of total endovascular repair with physician-modified fenestration (PM-F) or *in situ* fenestration (ISF) for patients suffering from aortic arch pathology with an ILVA in the present study.

## Methods

**Figure 1:**
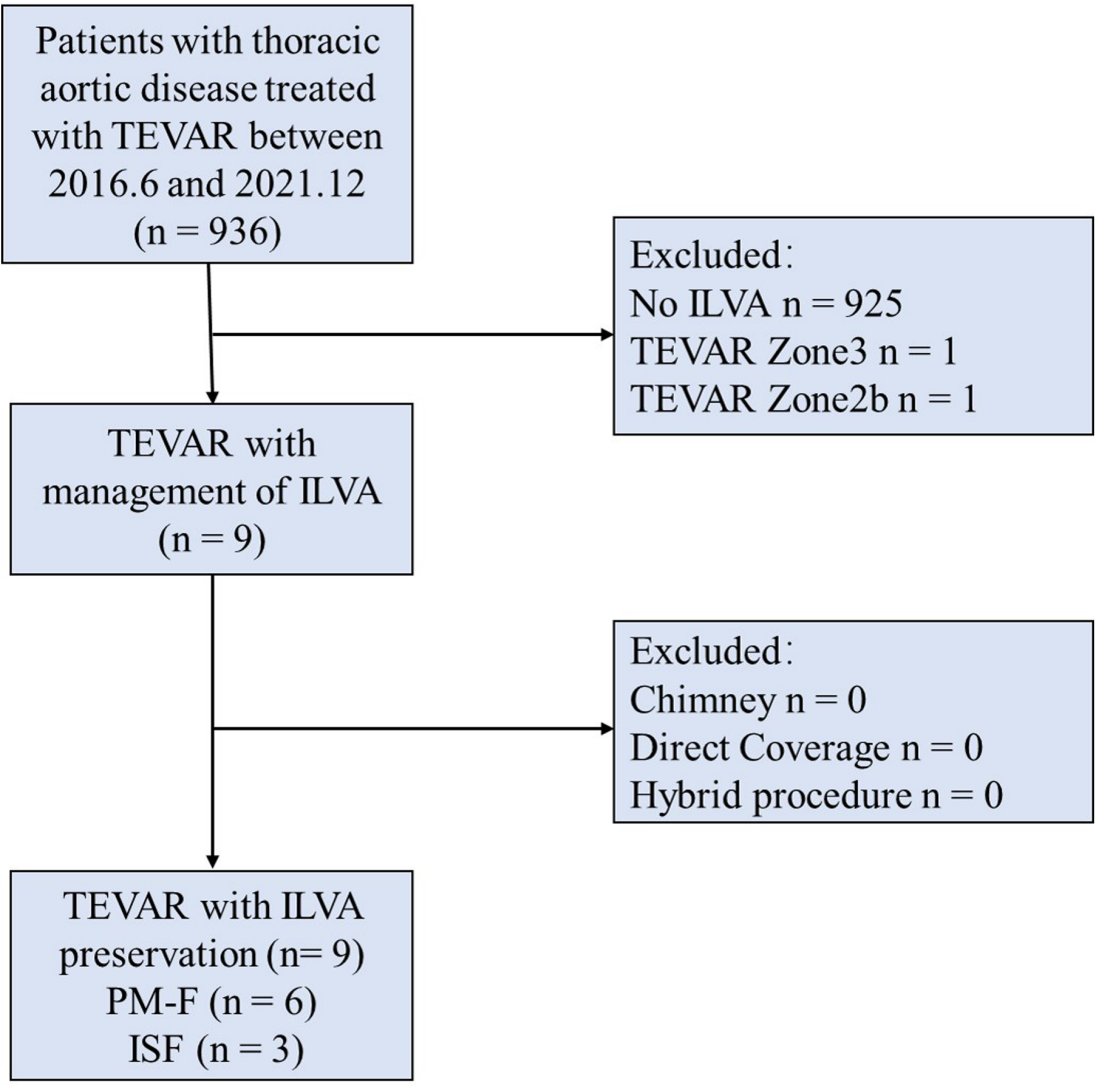
Consort diagram of thoracic aortic repairs (2017.6–2021.12; *n* = 936). TEVAR, thoracic endovascular aortic repair; ILVA, isolated left vertebral artery; PM-F, physician modified-fenestration; ISF, *in situ* fenestration.

This is a single-center, retrospective, observational cohort study. All the TAD patients treated in our center between June 2016 and December 2021 were retrospectively re-evaluated (Figure 1).

Inclusion criteria were as follows: (1) TAD patients with ILVA underwent total endovascular repair; (2) ILVAs were reconstructed with PM-F or ISF techniques. The indications of aortic disease intervention were deﬁned according to recommended clinical practice Guidelines of the European Society for Vascular Surgery (ESVS) (10). In our center, ILVA revascularization was performed in patients with a dominant ILVA or symmetric vertebral arteries and an incomplete circle of Willis. Extensive coverage of the aorta is also considered an indication for ILVA preservation to prevent spinal cord ischemia. The ILVA revascularization was not considered in patients with dominant right vertebral artery or a small ILVA (<2.0 mm in diameter). We did not perform open ILVA revascularization in our center.

Exclusion criteria were as follows: (1) Patients without ILVA; (2) Patients treated with open surgery or hybrid procedure; (3) Patients underwent endovascular repair without ILVA reconstruction (Zone 3 TEVAR or Zone 2b TEVAR or direct coverage); (4) ILVAs were reconstructed with other endovascular technique such as parallel stents.

The study was conducted in accordance with the Declaration of Helsinki. The study was approved by institutional ethics committee of our hospital (No. 20221434) and individual consent for this retrospective analysis was waived. We present the following article in accordance with the STROBE reporting guidelines.

All patients underwent preoperative computed tomography angiography (CTA) with 3-dimensional reconstructions on a workstation (QUARIUS WS, Terarecon Inc, Mateo, CA;). The stent-grafts were oversized 5%–10% for aortic dissections and 15%–20% for aortic aneurysms and penetrated ulcers. A landing zone of at least 1.5 cm away from proximal end of aortic lesions along the outer curvature of aortic arch was planned for either technique.

### Physician modified-fenestration for isolated left vertebral artery

Based on the three-dimension reconstruction, the information including the aortic diameters, the aortic arch angle, the branch vessel diameters, lengths, angles to the arch, clock positions, relative spatial relationships among the branches were taken into consideration to design the location of the fenestrations for ILVA. The strut-free area between the stent struts was preferred as the site of fenestration. The size of the fenestration was designed equal to or slightly smaller than the diameter of the ILVA origin. Then a circular fenestration was created with a cautery device or a knife. Two radiopaque markers were sewn onto the proximal and distal edge of the fenestration.

The main stent graft was introduced and rotated in the descending aorta to adjust the position of fenestration. The fenestration was oriented toward the ILVA by aligning the radiopaque markers with the origin of ILVA. Bridge stent implantation was preferred for aortic lesions not located on the lesser curvature. After full deployment of the stent graft, a bare metal stent of 3.5 mm to 5 mm in diameter was deployed (Supplementary Material, Figure 2). The bare stent used were balloon-expandable bare stent [Dynamic Renal (BIOTRONIK AG, Buelach, Switzerland) or Apollo (Microport, Shanghai, China)] or self-expandable bare stent [Pulsar-18 (BIOTRONIK AG, Buelach, Swizerland)].

**Figure 2:**
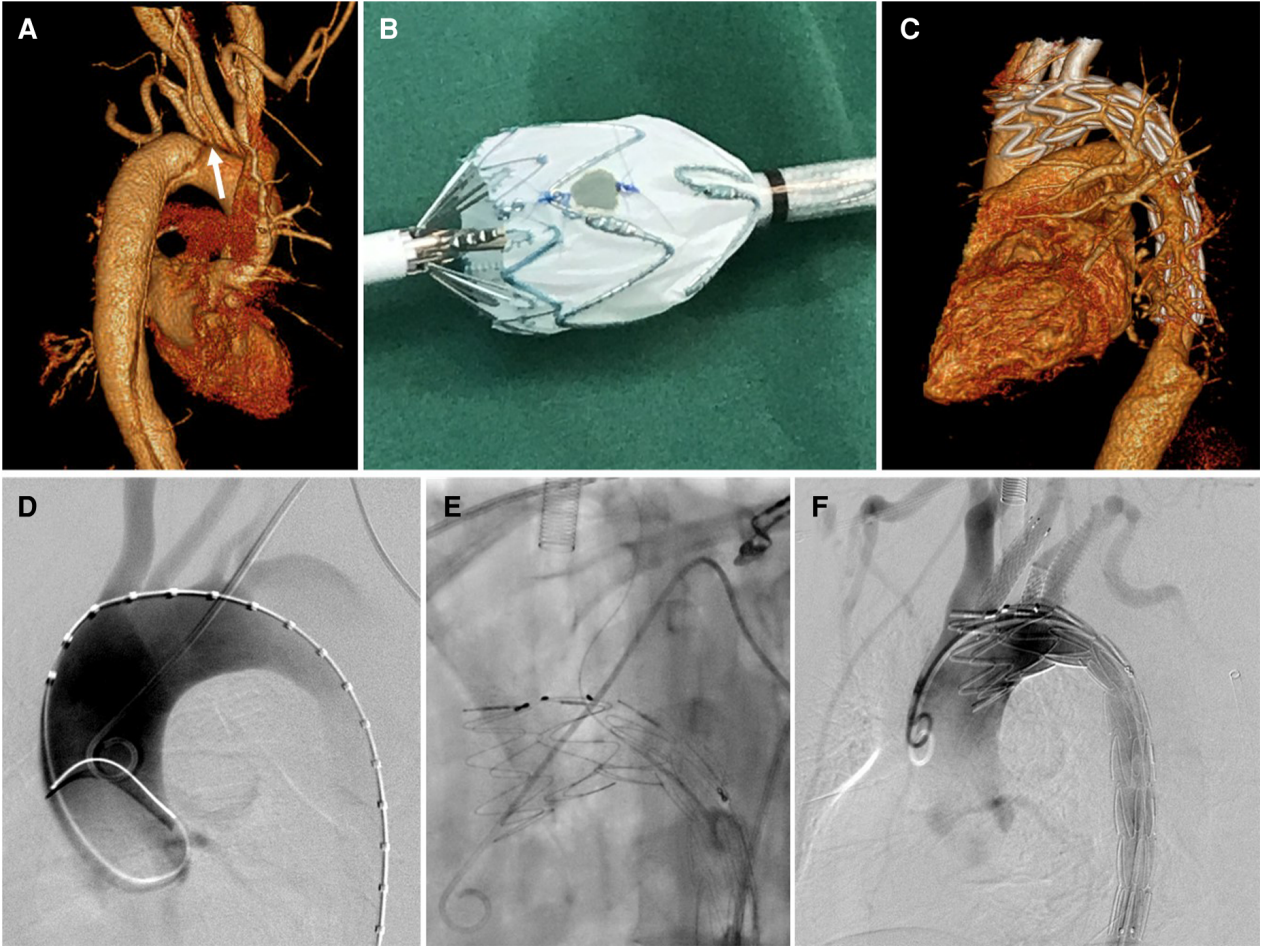
Physician modified-fenestration for isolated left vertebral artery. (**A**) Preoperative CTA of a type B aortic dissection demonstrating the ILVA (white arrow) from the distal aortic arch between the LCCA and the LSA; (**B**) A fenestration was made on the main body stent graft for preservation of ILVA on table; (**C**) follow up CTA showed patent target vessels and well excluded aortic dissection without endoleak; (**D**) intraoperative aortography showed a type B aortic dissection with a ILVA; (**E**) After full deployment of the stent graft, the ILVA was super-selected from the contralateral femoral access followed by bridging stent implantation; (**F**) Final aortography demonstrated complete exclusion of aortic dissection and patent aortic arch branch arteries, with bridging stent-grafts in the LCCA, ILVA, and LSA.

### *In situ* fenestration for isolated left vertebral artery

**Figure 3:**
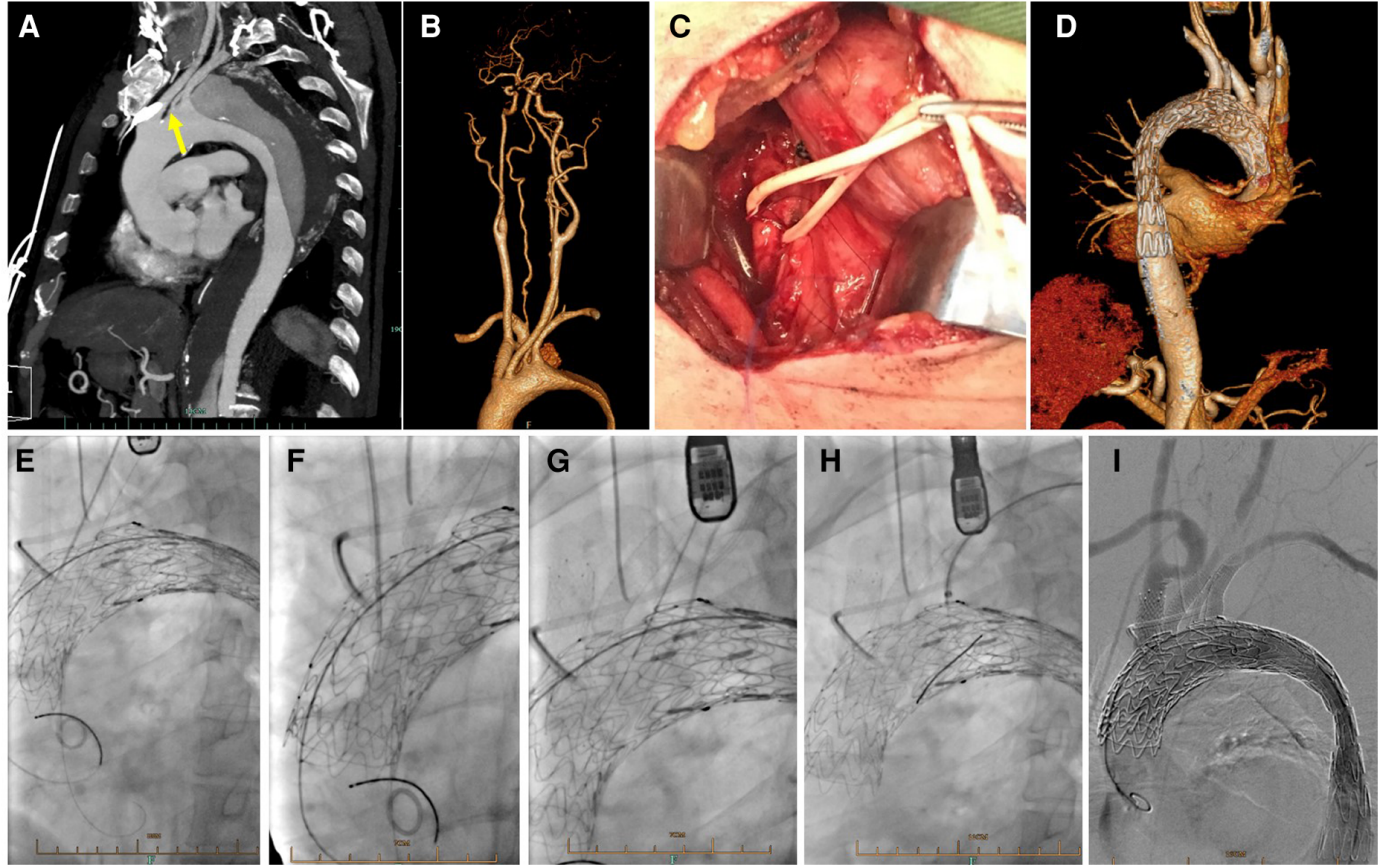
*In situ* fenestration for isolated left vertebral artery. (**A**) Preoperative CTA showed a type B aortic dissection with ILVA (yellow arrow); (**B**) The cervicle contrast-enhanced CTA demonstrated dominant ILVA and incomplete circle of Willis; (**C**) During procedure, the ILVA was exposed with a supraclavicular incision; (**D**) Follow up CTA showed well exclusion of aortic dissection and patent supra aortic branches; Needle-assisted *in situ* fenestration followed by bridging stents placement for reconstruction of LCCA (**E**), BCT (**F**), ILVA (**G**) and LSA (**H**) after deployment of stent graft; (**I**) Complete aortography demonstrated excluded aortic dissection and patent branch vessels.

An incision was made at the interval of sternocleidomastoid branches, one attaching on manubrium while the other attaching on proximal part of the clavicle for exposure of ILVA. Then a 6F short sheath was introduced into ILVA. After full deployment of the stent graft, a liver biopsy needle (18 gauge/30 cm, BARD) was introduced *via* the short sheath, and advanced to the ostium of ILVA. After the tip was adjusted perpendicular to the greater curvature of the aortic stent graft, the membrane of the stent graft was penetrated to make a fenestration. The fenestration was dilated with a 3- or 4 mm balloon and then a 4- to 5 mm bare metal stent was deployed as a bridge stent. Post-dilation with a 3- or 4 mm balloon was then conducted routinely (Figure 3, Supplementary Material).

In our center, the left subclavian artery (LSA), brachiocephalic trunk (BCT) and LCCA were generally reconstructed by ISF technique if required (11), regardless of the technique used for ILVA reconstruction. Chimney technique was only considered when the supra aortic arch vessel was accidently partially covered by thoracic stent graft. The preferred subsequence for reconstruction was LCCA, BCT, ILVA and LSA. For patients requiring fenestration of the LSA, an 8F angle-adjustable sheath (Lifetech, Inc., Shenzhen, China) was exchanged from the left brachial artery access. Then an adjustable needle catheter (12) was advanced *via* the sheath and punctured the aortic stent graft at an as perpendicular as possible angle. After sequential ballooning with 4-, 8-, and 10 mm balloon, a bridging stent with appropriate sizes was implanted. For patients requiring fenestration of both the LCCA and LSA, a liver biopsy needle (18 gauge/30 cm, BARD) was used to create the LCCA fenestration first. The balloon dilatation and stent implantation of the LCCA were similar to the procedures of LSA fenestration. For patients requiring fenestration of LCCA, BCT and LSA (13), LCCA was reconstructed first followed by BCT reconstruction. The cardiopulmonary bypass was applied between coverage of LCCA and BCT and successful reconstruction of the two TVs (13).

After the procedure, mono-antiplatelet therapy (aspirin, 100 mg/day) was prescribed for long-term therapy.

### Follow up

Demographic, anatomical, intra-operative, and post-operative data were recorded. All patients underwent computerized tomography (CT) scan pre-operatively and before discharge. The follow up protocol included CT scan at 1, 3, 6, and 12 months and yearly thereafter (14). The follow up clinical data was obtained during patient visits to the hospital, other hospital stays, or by telephone interview.

### Definition and outcomes

The results were presented according to the guidelines for reporting standards in TEVAR (15). Technical success was defined as successful deployment of all stent grafts with patent TVs and exclusion of the lesion in the absence of surgical conversion to open repair or death at 24 h or less without type I or III endoleak in the completion angiogram. Major adverse events (MAEs) included all-cause mortality, major stroke, paraplegia, myocardial infarction, respiratory failure, renal function decline or new-onset dialysis, bowel ischemia, and other major complications. Target vessel stenosis less than 50% was defined as patency. Short term was defined as the first 30 postoperative days. The follow up index was defined as the ratio between the investigated follow-up period and the theoretically possible follow-up period up to the pre-specified study end date (16). Classification of the vertebral artery variable origin was defined according to Lazaridis' report (4). Aortic arch aneurysms were classified according to Cooley's report (17) based on the extent of the aneurysm and the repair. The primary endpoints were all-cause mortality and neurologic new symptoms. The secondary endpoints were ILVA patency rate, endoleak and other complications.

### Statistical analysis

Statistical analysis was performed using SPSS software (version 19.0; SPSS, Inc., Chicago, IL, United States). Continuous variables were summarized as means ± standard deviations if normally distributed, and as median and range if not. Categorical variables were expressed as count and percentage.

## Results

Between June 2016 and December 2021, a total of 9 TAD patients (88.9% male with median age of 60.0 years, range: 38.0–76.0) underwent TEVAR and ILVA reconstruction. According to the proposed classification (4), ILVA presented with the LA2.2 configuration in all patients. Left VA dominance, right VA dominance and symmetric VA were found in two (22.2%), two (22.2%) and five (55.6%) patients. In eight (88.9%) patients, the ILVAs entered the circle of Willis to form the basilar artery. In eight (88.9%) patients, the ILVAs sent off the posterior-inferior cerebellar artery. Demographic characteristics and baseline clinical data are detailed in Table 1.

**Table 1:** Demographics and baseline clinical characteristics (*n* = 9).

Variable	*N* (%) or median (range)
Age [years, median (range)]	60.0 (38.0–76.0)
**Sex**	
Male	8 (88.9%)
Female	1 (11.1%)
**Comorbidities**	
Hypertension	6 (66.7%)
Hyperlipidemia	0
COPD	1 (11.1%)
Diabetes	2 (22.2%)
Coronary artery disease	1 (11.1%)
Myocardial infraction	0
Congestive heart failure	0
Previous stroke	1 (11.1%)
Peripheral artery disease	0
Renal insufficiency	0
Renal failure	0
Previous aortic surgery	0
History of tumor	1 (2.6%)
Cigarette Use	7 (77.8%)
**ASA classification**	
II	5 (55.6%)
III	4 (44.4%)
**Pathology**	
Type B aortic dissection	4 (44.4%)
Acute	2 (22.2%)
Chronic	2 (22.2%)
Thoracic aortic aneurysm	4 (44.4%)
PAU	1 (11.1%)
**Anatomic features**	
ILVA configuration	
LA2.2^a^	9 (100.0%)
Left vertebral artery dominance	2 (22.2%)
Right vertebral artery dominance	2 (22.2%)
Symmetric vertebral artery	5 (55.6%)
ILVA diameter, mm	3.4 (3.0–4.2)
Right vertebral artery diameter, mm	4.0 (3.0–5.1)
Complete circle of Willis	2 (22.2%)
ILVA entering the circle of Willis to form the basilar artery	8 (88.9%)
ILVA sending off the posterior–inferior cerebellar artery	8(88.9%)

COPD, chronic obstructive pulmonary disease; PAU, Penetrating atherosclerotic ulcer; ILVA, Isolated left vertebral artery.

^a^ The ILVA configuration classification was based on Lazaridis’ report (*Surg Radiol Anat*. 2018; **40**:779–97).

All patients received TEVAR with aortic arch branches reconstruction. The technical success rate was 100%. Two TVs (LSA and ILVA) were reconstructed in 6 patients, three TVs (LSA, ILVA, and LCCA) in 2 patients and four TVs (LSA, ILVA, LCCA and BCT) in one patient. Totally, 22 TVs were successfully reconstructed (ISF used for 15 TVs, PM-F for 6 TVs and Chimney technique for one TV) and 19 bridging stents (9 covered stents and 10 bare metal stents) were placed in 19 TVs. Among the nine preserved ILVAs, PM-F technique were used for 6 TVs while ISF technique were used for 3 TVs. Six bare metal stents were placed in 6 ILVAs. In three ILVAs preserved with PM-F, stent was not deployed. The main body stent grafts used were the Ankura (Lifetech, Shenzhen, China; *n* = 5), Valiant (Medtronic, Inc, Minneapolis, MN, United States; *n* = 3) and TAG (Gore, WL Gore & Associates, Flagstaff, AZ, United States; *n* = 1) devices. The median procedure time was 170.0 (range: 110.0–375.0) min and the volume of contrast material was 105.0 (range: 90.0–200.0) ml. The median hospitalization was 11.0 (range: 7–24) days and the median length of stay in ICU after the operation was 0 (range: 0–1) days (Table 2).

**Table 2:** Procedure details.

Variable	*N* (%) or median (range)
Emergency operation	0
Technical success	9 (100%)
Procedure time, minutes	170.0 (110.0–375.0)
Volume of contrast material, ml	105.0 (90.0–200.0)
Length of thoracic aortic endografts, mm	160.0 (150.0–200.0)
Diameter of thoracic aortic endografts, mm	32.0 (30.0–40.0)
**Proximal landing zone**	
Z2	6 (66.7%)
Z1	2 (22.2%)
Z0	1 (11.1%)
**Distal landing zone**	
Z5	9 (100%)
**Total target vessel**	
LSA	9
ILVA	9
LCCA	3
BCT	1
No. of bridging stents per patient	2.0 (1.0–4.0)
**Technique for ILVA reconstruction**	
ISF	3 (33.3%)
Pre-fenestration	6 (66.6%)
Stent placement in ILVA, patient	6 (66.7%)
Covered stent	0
Bare metal stent	6 (66.7%)
Length of ILVA stent, mm	26.0 (19.0–40.0)
Diameter of ILVA Stent, mm	4.25 (3.5–5.0)
Length of stay, days	11.0 (7.0–24.0)
Length of ICU stay, days	0 (0–1.0)

LSA, left subclavian artery; ILVA, isolated left vertebral artery; LCCA, left common carotid artery; BCT, brachiocephalic trunk; ICU, intensive care unit.

There was no early mortality within 30 days after procedure. No aortic rupture, major stroke, spinal cord injury, acute kidney injury, renal failure and other major adverse event was observed. No endoleak was detected *via* 30-day follow up CTA. No patient received reintervention. All TVs were patent without occlusion/stenosis or bridging stent migration.

The median follow up was 38.0 (range: 1.0–66.0) months. All patients were followed up and the mean follow up index was 1.0 ± 0.0. During follow up period, one death occurred at 56 months resulting in a follow up mortality of 11.1% (1/9). The reason for death cannot be identified. Out of the 22 successfully revascularized TVs, 20 TVs remained patent while 2 ILVAs were found occluded. The ILVAs occlusion occurred at 6 months and 7 months, respectively. The ILVA patency rate was 77.8% (7/9). Fortunately, these patients were all asymptomatic. Hence, further reintervention was not needed. No other major adverse event including aortic rupture, major stroke or spinal cord injury was observed during follow up. No endoleak was detected *via* follow up CTA. No significant stenosis, kink, fracture and migration of branch stents were observed. No patient received reintervention (Table 3). The follow-up CTA indicated that all the patients exhibited a reduction in the diameter of the aneurysm and the thrombosed false lumen. The median maximum aortic diameters were 36.0 (range: 25.0–66.0) mm preoperatively and 30.2 (range: 22.0–63.0) mm at last follow up, respectively (*p* = .001).

**Table 3:** Follow up outcomes.

Variable	*N* (%) or median (range)
Follow-up, months	38.0 (1.0–66.0)
FU mortality	1 (11.1%)
FU MAE	3 (33.3%)^a^
**FU endoleak**	
Type I	0
Type II	0
Type III	0
FU Re-intervention	0
FU TV instability^b^	2 (22.2%)
**Branch vessels**	
Branch occlusion/stenosis	2 (22.2%)
Bridging stent migration	0
ILVA occlusion/stenosis	2 (22.2%)

MAE, major adverse event; TV, target vessel; ILVA, isolated left vertebral artery; FU, follow up.

^a^ Follow up MAE included one death and two ILVA occlusions taking place during follow up period.

^b^ TV instability means composite end point used to define any death or rupture related to side branch complication (e.g., endoleak, rupture) or any secondary intervention indicated to treat a branch-related complication, including endoleak, disconnection, kink, stenosis, occlusion, or rupture.

## Discussion

ILVA is not a rare aortic arch branch variation, which is more common in TAD patients (1). The presence of an ILVA has significant impact on the choice of aortic arch reconstruction techniques and cerebral protection methods (18). Current literature reported several options such as total open surgery (18), hybrid procedure (9, 19) and parallel stents technique (20) to deal with aortic arch lesions and ILVAs. However, there was no consensus on the indication and strategy for ILVA reconstruction to date, as the relevant studies were scarce.

ILVA can enter into basilar artery and terminate at posterior inferior cerebellar artery (PICA), which can supply the brainstem and cerebellum. It is necessary to manage the ILVA better to prevent posterior circulation ischemia, stroke and spinal cord injury, instead of direct coverage. According to Ding and his colleague's experience (21), preservation of ILVA was favored, if ILVA was dominant, or if bilateral vertebral artery was symmetric with an incomplete circle of Willis. Differently, Yang and his colleague (9) held that all ILVA should be preserved if possible based on the fact that the prevalence of complete circle of Willis was 42% in western population and only 27% in Chinese population (8, 22). And, Piffaretti and his colleagues held that reconstruction of a nondominant ILVA can help reduce the potential risk of spinal cord ischemia in patients with additional risk factors such as previous extensive aortic coverage (19). We favored a positive strategy to preserve ILVA for a high incidence (73%) of an incomplete circle of Willis in the Chinese population (8). In our clinical practice, we performed ILVA revascularization in patients with a dominant ILVA or symmetric vertebral arteries and an incomplete circle of Willis. Extensive coverage of the aorta is also considered an indication for ILVA preservation to prevent spinal cord ischemia. The ILVA revascularization was not performed in patients with dominant right vertebral artery or a small ILVA (<2.0 mm in diameter).

Currently, during open surgery and hybrid procedure, ILVA transposition combined with LSA transposition seemed a feasible and reliable approach. The limited evidence from recently published case series showed high technical success rate and high long-term ILVA patency rate (9, 19). During perioperative and follow up period, there was no neurological complications. However, the small sample size and short follow-up limited the quality of the evidence. An endovascular approach was worth trying to provide a less invasive and more expeditious method which can be completed in one stage. Table 4 (9, 18, 19, 23–25) has summarized the results of various techniques for ILVA revascularization from currently published case series (number of patients >1) to date.

**Table 4:** Techniques for preservation of isolated left vertebral artery: Literature summary^a^.

Author	Year	No.	Disease	Treatment for aortic disease	ILVA preservation	Success rate	Early outcomes	FU (moths)	FU outcomes	FU ILVA patency
Suzuki et al. (24)	2006	10	TAA(*n* = 8) TAD(*n* = 2)	Open repair	^b^en-bloc technique (*n* = 1); Transposition (*n* = 9)	100%	None	NA	NA	NA
Qi et al. (23)	2013	21	TAD	Open repair	en-bloc technique (*n* = 12); Transposition (*n* = 9).	100%	2 spinal cord injury; 1 transient neurologic deficit; 1 acute renal failure.	58 ± 16	1 late death	NA
Zhu et al. (18)	2015	3	TAD	Open repair	Transposition	100%	None	44 ± 19	1 patient underwent TEVAR for descending aortic dissection	100%
Piffaretti et al. (19)	2020	6	TAA(*n* = 3) TAD(*n* = 3)	Open repair (*n* = 4) TEVAR(*n* = 2)	Transposition	100%	1 horner's syndrome; 1 respiratory insufficiency	Mean 4.5	1 death at 4 month	100%
Yang et al. (9)	2021	13	TAA(*n* = 2) TAD(*n* = 8) IMH(*n* = 2) PAU(*n* = 1)	TEVAR	Transposition	100%	1 contrast induced acute kidney injury; 1 incision hematoma; 1 acute left-lower-limb ischemia	Mean 22	None	100%
Zhang et al. (25)	2022	67	TAA(*n* = 12) TAD(*n* = 43) IMH(*n* = 7) PSA (*n* = 5)	TEVAR	Chimney (*n* = 28); Pre-fenestration (*n* = 24) Transposition (*n* = 15)	100%	9 Ia endoleak	64 ± 4	7 neurologic new symptoms; 9 Ia endoleak; 5 mild-dizziness	^c^97%

TAA, thoracic aortic aneurysm; TAD, thoracic aortic dissection; ILVA, isolated left vertebral artery; FU, follow up; NA, not available; TEVAR, Thoracic Endovascular Aortic Repair; IMH, intramural hematoma; PAU, penetrating atherosclerotic ulcer; PSA, pseudoaneurysm.

^a^ Only case series (*n* > 1) were included.

^b^ En-bloc technique means a single aortic patch containing the origin of the ILVA and the left subclavian artery or left common carotid artery was anastomosed to the prosthetic graft.

^c^ Two patients in ILVA transposition group were found to have an occluded ILVA.

Parallel grafts such as the chimney technique has been used for preservation of ILVA, implying a feasible alternative with encouraging short-term results (20). However, the risk of type Ia endoleaks through the gutters and uncertainty regarding the long-term patency of artery remain a concern (26). PM-F (27) and ISF (11) has been used in supra-aortic branches reconstruction. The published literature showed that these two techniques were promising and reliable methods excluding the lesions and preserving the target vessels. To date, these two techniques has not been reported for preservation of ILVA during TEVAR. Based on our experience of performing PM-F and ISF for preservation of other target vessels, less invasive and total endovascular method was provided for the patients in this case series. For patients requiring reconstruction of both the ILVA and LSA, PM-F technique was preferred as the cervical incision can be avoid. For patients requiring reconstruction of more supra-arch branches, ISF was preferred as ILVA can be exposed simultaneously during exposing LCCA in which cervical incision was inevitable. During perioperative period, the technical success rate was high without major adverse events and procedure-related vessel injury, nerve injury, chyle leakage and lymphatic leakage in this case series. All target vessels were patent during short-term period (<30 days). Currently, both laser fenestration technique and needle fenestration technique had been reported to be used during *in situ* fenestration procedure for treatment of aortic arch disease with favorable outcomes (11, 28). We preserved the ILVA using needle assisted fenestration technique in these patents for we were familiar with needle fenestration procedure. Laser fenestration technique could also be considered as a potential adjunct for ILVA revascularization based on experience of different centers.

Some drawbacks still existed and raised some concern during application of these two techniques. For PM-F, massive pre-operative measurements and accurate deployment were needed for better alignment of fenestrations to the ostium of target vessels. The process of stent graft modification would extend the operation time. The modification procedure of removing a part of membrane may impact the integrity and durability of the stent graft. And the short junction between the main body stent graft and the bridging stent may increase the risk of type III endoleak. For ISF, the manipulation is really technical demanding. Vessel injury may occur during fenestration process. The supra arch arteries and cerebral blood supply has to be blocked before successful fenestration which could increase the risk of cerebral ischemia. And the concern of high risk of type III endoleak still existed for short combination of the main body stent graft and the bridging stents (29). However, our initial experience showed that favorable short-term results can be achieved without neurological deficits and other major complications.

At present, limited data on ILVA transposition during open surgery and hybrid procedure has been published showing satisfactory patency rate during short-term follow up period (9, 18, 19). But the interpretation of the results should be careful as the number of patients was small and the studies were single-center retrospective case series showing high risk of bias. In our study, patency rate was favorable during postoperative period. However, the patency rate dramatically decreased around 6 months follow up. This initial experience implied unsatisfied long-term patency (77.8%) of ILVA reconstructed by PM-F or ISF. However, it should be note that the mean diameter of ILVA in this study (3.4 mm) was smaller compared with the figure of 5.1 mm in the current literature (9). On the other hand, there was no cerebral infarction or SCI observed in the two patients with ILVA occlusion. It is difficult to determine why patients do not have severe posterior circulation ischemia related symptoms or spinal cord ischemia after vertebral artery occlusion. The plausible explanation was that ILVA occlusion after ILVA reconstruction is a relatively slow process (around 6 months). Collateral pathways can develop and compensate for posterior circulation ischemia during that process, which is different from acute ischemia caused by direct coverage without collateral pathways compensation. Nonetheless, as current evidence was really scarce, it may be reasonable to preserve ILVA to decrease risk of cerebral ischemia and spinal cord injury. The endovascular technique is a worthwhile alternative with less invasiveness.

### Limitations

There are some limitations of this study. This is a single center, retrospective observational study with a relatively small number of patients and relatively shorter follow-up period. In addition, it lacks control groups. Further, the surgeon experience may impact the results of the procedure.

## Conclusions

PM-F or ISF for ILVA preservation was feasible, safe, and effective. Issues on indication and technical strategy for ILVA preservation should be better discussed and clarified. And, the patency of ILVA preserved by PM-F or ISF should be further improved.

## Data Availability

The original contributions presented in the study are included in the article/Supplementary Material, further inquiries can be directed to the corresponding authors.
